# Twenty-eight Pregnant Women with Cardiac Diseases: A Review of Maternal and Fetal Outcomes in Pakistan

**DOI:** 10.7759/cureus.6034

**Published:** 2019-10-30

**Authors:** Shoaibunnisa Soomro, Raj Kumar, Altaf A Shaikh

**Affiliations:** 1 Obstetrics and Gynecology, Ghulam Muhammad Mahar Medical College and Hospital, Sukkur, PAK; 2 Cardiology, Ghulam Muhammad Mahar Medical College and Hospital, Sukkur, PAK; 3 Internal Medicine, Ghulam Muhammad Mahar Medical College and Hospital, Sukkur, PAK

**Keywords:** pregnancy, pregnancy complications, cardiomyopathy in pregnancy, pakistan, pakistan

## Abstract

Introduction

In the western world, 0.2%-4% of pregnancies are complicated by heart diseases. The United Kingdom Obstetric Surveillance System (UKOSS) conducted a study on acute myocardial infarction (MI) in pregnant women. It estimated an incidence of 0.7 cases of acute MI per 100,000 pregnancies. In this study, we review the maternal and fetal outcomes of pregnant women with a cardiac disorder.

Methods

This retrospective analysis included all maternal records of the women registered in the obstetrics and gynecology department of Ghulam Muhammad Meher Medical Hospital, Sukkur, from January 1 to December 31, 2018. The study was approved by the institutional review board.

Results

The most common cardiac disease among our patients was peripartum cardiomyopathy (n=12; 42.9%) followed by rheumatic heart disease in nine (32.1%) women. The rate of maternal mortality was 14.2% (n=4). There were eight (28.6%) cases of intrauterine devices (IUDs) and the remaining 20 (71.4%) babies were born alive and healthy.

Conclusion

The prevalence of cardiac diseases in pregnancy in Pakistan is comparable to that in our neighboring countries. These cardiac diseases are responsible for fetal and maternal adverse outcomes.

## Introduction

In the western world, 0.2%-4% of pregnancies are complicated by heart diseases [1]. The United Kingdom Obstetric Surveillance System (UKOSS) conducted a study on acute myocardial infarction (MI) in pregnant women. It estimated an incidence of 0.7 cases of acute MI per 100,000 pregnancies [2]. Acquired heart diseases are becoming more common in pregnancy due to older age at the time of first pregnancy and higher frequency of predisposing cardiovascular (CV) risk factors, including hypertension (HTN), diabetes mellitus (DM), and obesity [3]. Another important reason is that the advancements in medical care have allowed more women with cardiac diseases to conceive and carry pregnancy till term [4].

In developing countries, rheumatic heart diseases (RHD) constitute as many as 56%-89% CV co-morbidities during the antepartum period [5]. In an Indian study, 69% of cases of CV co-morbidity during pregnancy were due to RHD [6]. In Pakistan, 1% of pregnancies are complicated by cardiac diseases and as with India, 69% are caused by RHD [7].

Peripartum cardiomyopathy (PPCM) is the idiopathic failure of the myocardium. It occurs during the last month of pregnancy and may persist until five to six months of the postpartum period. Some women may develop signs of PPCM right after delivery or within a month postpartum rather than during the pregnancy [8]. The incidence of PPCM has been reported from many countries and has been highest in Nigeria (1/102 deliveries) and lowest in Japan (1/15,533 births) [9]. In Pakistan, younger women with higher parity and pregnancy-induced hypertension (HTN) are at high risk of PPCM [10].

Despite this, there are very few studies available in Pakistan focused especially on heart diseases in pregnancy, including clinical symptoms and radiological findings, in any part of the country. The aim of our study was to highlight the clinical and radiological findings and fetal and maternal outcomes in such patients so high-risk patients should be identified and more-focused treatment could be given to them.

## Materials and methods

This retrospective analysis included all maternal records (n=2,282) of the women registered in the obstetrics and gynecology department of Ghulam Muhammad Meher Medical Hospital, Sukkur, from January 1 to December 31, 2018. The study was approved by the institutional review board.

Among all obstetric records, women with CV co-morbidity were identified and their patient records were extracted for review. Their type of CV co-morbidity, chest X-ray findings, electrocardiogram (ECG) and echocardiography (echo) findings, and maternal and fetal outcomes were included in this report. Data were presented as a tabulated form, and frequency and percentages were calculated.

## Results

During the study period, 2,282 obstetric cases were booked for delivery in the department. CV co-morbidity was established in 28 (1.2%) cases. Out of these, 21 (75%) were diagnosed with CV co-morbidity during pregnancy and the remaining 7 (25%) had chronic CV co-morbidity. The most common cardiac disease among our patients was peripartum cardiomyopathy (n=12; 42.9%) followed by rheumatic heart disease in nine (32.1%) women. Eighteen (64.4%) women out of 28 were unbooked.

The rate of maternal mortality was 14.2% (n=4). All four women had PPCM. The baby of only one of these four women was born alive and the remaining three were intrauterine deaths (IUD). There were eight (28.6%) cases of IUD and the remaining 20 (71.4%) babies were born alive and healthy. Among these cases of IUD, four mothers had PPCM, two had RHD, one had pre-eclampsia, and one had ischemic heart disease (IHD).

A detailed report of the chest X-ray, ECG, and echo findings, along with the maternal and fetal outcomes of these cases are shown in Table 1.

**Table 1:** Cardiovascular co-morbidity, chest x-ray, ECG, and echo findings, and maternal and fetal outcomes of pregnant women Abbreviations: AF, Atrial Flutter; CT, Cardiothoracic; EF, Ejection Fraction; LA, Left Atrium; LAD, Left Axis Deviation; LHB, Left Heart Border; LV, Left Ventricle; LVH, Left Ventricular Hypertrophy; MC, Mitral Cusp; MR, Mitral Regurgitation; MS, Mitral Stenosis; RA, Right Atrium; RAD, Right Axis Deviation; RHB, Right Heart Border; RV, Right Ventricle; RVH, Right Ventricular Hypertrophy; TR, Tricuspid Regurgitation

S. No	Type of Cardiac Disease	ECG Finding	X-ray Findings	Echo Findings	Maternal Outcome	Fetal Outcome
1	Peripartum cardiomyopathy	LA + LV enlargement	Increased CT ratio, increased pulmonary congestion	Dilated all 4 chambers, global LV, hyperkinesias	Alive	Alive
2	Pre-eclampsia	LVH + LA enlargement	Increased CT ratio, increased pulmonary congestion, upper tube diversion	EF: 35-40%, dilated all 4 chambers, moderate generalized depressed LV function	Alive	IUD
3	Rheumatic heart disease	RAD, RVH, LA/LV enlargement	Straightening of LHB with double contour shadow on RHB, upper lobe diversion	Severe MS, mild MR, EF: 70%	Alive	Alive
4	Mitral stenosis	RAD, RVH, LA enlargement	Straightening of LHB with double contour shadow on RHB, upper lobe diversion	Severe MS, calcified valves, normal size LV, normal function	Alive	Alive
5	Acute pulmonary edema	P-mitrale, dilated Ram RV, depressed RV function	Straightening of LHB, double contour shadow on RHB, upper lobe diversion, batwing appearance, severe pulmonary congestion	Severe mitral RAD stenosis, dilated LA, EF: 70%	Alive	Alive
6	Rheumatic heart disease	P-mitrale, dilated LV, severe LV dysfunction	Increased CT ratio with bilateral obliteration of costophrenic angles, severe pulmonary congestion	EF: 25%, thickened mitral valve, moderate MS and severe MR	Alive	IUD
7	Peripartum cardiomyopathy	LA + LV enlargement	Increased CT ratio with pulmonary congestion	Dilated all 4 chambers with severe generalized LV dysfunction, EF: 25-35%	Alive	Alive
8	Peripartum cardiomyopathy	P-mitrale, LA + LV enlargement	Increased CT ratio with pulmonary congestion, obliterated both costophrenic angles, batwing appearance	Dilated LA & LV, severe LV dysfunction	Alive	Alive
9	Peripartum cardiomyopathy	AF	Increased CT ratio with pulmonary congestion, mild pleural effusion on right side	Dilated LV, severe LV dysfunction, Dilated LA	Alive	Alive
10	Peripartum cardiomyopathy	LVH & LA enlargement + LAD	Dilated LA & LV with increased vasoconstriction, mild pleural effusion on right side	Dilated LV with global hyperkinesias, EF:25-30%	Alive	Alive
11	Peripartum cardiomyopathy	AF	Normal CT ratio with prominent aortic knuckle	Concentric hypertrophied LV with normal EF	Alive	Alive
12	Peripartum cardiomyopathy	LA & LV enlargement	Dilated LA & LV, double contour shadow on RHB, right-sided pleural effusion	EF: 20-25%, Dilated LF with generalized hypokinesia	Maternal death	IUD
13	Rheumatic heart disease	P-mitrale, LVH, LAD	Enlarged CT ratio with increased vascularity, batwing appearance	Generalized LV dysfunction, EF: 20-25%	Alive	Alive
14	Ischemic heart disease	Poor R wave in V1-V6, LAD	Increased CT ratio with pulmonary congestion	Mildly dilated LV with apical hypokinesia, EF: 35-40%	Alive	IUD
15	Peripartum cardiomyopathy	LVH + LAD + AF	Increased CT ratio with increased vascularity, dilated pulmonary arteries	Dilated LV, gen. LV dysfunction, EF: 20-25%	Maternal death	IUD
16	Cardiogenic shock	LVH + LAD +AF	Increased CT ratio with increased vascularity with upper lobe diversion	Dilated LV, severe gen. LV dysfunction. Apical LV clot	Alive	Alive
17	Rheumatic heart disease	LA & LV enlargement	Increased CT ratio, double contour shadow on right side	Severe MS and moderate MR, dilated LA & LV with normal function	Alive	IUD
18	Rheumatic heart disease	LA enlargement + RAD	Increased CT ratio with straightening of LHB + venous congestion + LA enlargement	Severe MS with pliable valves	Alive	Alive
19	Peripartum cardiomyopathy	LA enlargement with Poor R in anterior leads	Increased CT ratio with pulmonary congestion + mild pleural effusion	Dilated LV with severely depressed function. EF: 30%	Maternal Death	IUD
20	Peripartum cardiomyopathy	P-mitrale +LVH + Poor R in anterior leads	Increased CT ratio with pulmonary congestion	EF: 20-25%	Alive	IUD
21	Rheumatic heart disease	P-mitrale + RAD	Straightening of LHB, double contour shadow on RHB, upper lobe diversion	Severe MS with calcified MV & mild MR	Alive	Alive
22	Primary pulmonary hypertension	RAD + RVH + P-mitrale	Dilated pulmonary artery, elongated lung shadow	Moderate TR + Moderate dilated RA and RV	Alive	Alive
23	Rheumatic heart disease	Within normal limits	Within normal limits	Relapsing AML with thickened MC leaflets (No MS)	Alive	Alive
24	Rheumatic heart disease	P-mitrale + RVH	Increased CT ratio with double density on RHB	Moderate MS + Moderate MR, Dilated both LA & LV	Alive	Alive
25	Peripartum Cardiomyopathy	LVH + Poor R in anterior leads	Increased CT ratio with pulmonary congestion, obliteration of costophrenic angles	Dilated LA & LV with severe LV dysfunction.	Alive	Alive
26	Peripartum cardiomyopathy	LVH + LAD	Increased CT ratio with pulmonary congestion	Dilated LV with severely depressed function. EF: 25%	Maternal death	Alive
27	Rheumatic heart disease	P-mitrale + RVH	Straightening of LHB, double contour shadow on RHB, increased venous congestion	Dilated both LA & LV	Alive	Alive
28	Aortic stenosis	No specific changes	Increased CT ratio with straightening of LHB, prosthetic valve shadow on left sternal border	Prosthetic mitral and aortic valves with hypertrophy, normal functioning LV	Alive	Alive

## Discussion

In our study, the prevalence of cardiac disease in pregnancy was 1.22%. This was consistent with a previous regional study conducted in India [6] and Pakistan [7]. Cardiac disease is the most common non-obstetric maternal death [11].

The most common cardiac disease reported in our study was cardiomyopathy. Twelve out of 28 patients were diagnosed with some form of cardiomyopathy. Peripartum cardiomyopathy is more common generally in a month before or after partum, as opposed to dilated cardiomyopathy, which already presents itself by the second trimester. Mortality in peripartum cardiomyopathy varies from <2% to 50% [11]. Heart failure and arrhythmia are the most common complications associated with cardiomyopathy in pregnancy [11]. There are various risk factors, such as obesity, diabetes, hypertension, and stress, during pregnancy, which is responsible for the increase in cardiomyopathy. Increased maternal age is also a known risk factor [6].

The second most common cardiac disease reported in our study was rheumatic disease. Unlike our study, rheumatic heart disease remains the number one worldwide cause of maternal cardiac complications in pregnancy. Since symptoms of rheumatic fever typically do not present until the fourth or fifth decade, the pathophysiology changes associated with pregnancy may cause as many as 25% of these women to first experience symptoms during pregnancy [12].

The maternal mortality in our study was 14.6%. This was slightly low as compared to that reported in our neighboring country of India [6] but, still, attention should be paid to this considerably high mortality rate when examined on its own.

While our study adds to the limited data available in Pakistan related to cardiac disease in pregnancy, it has its limitations. First, since this was not a long-term prospective study, fetal outcomes and implications on maternal outcomes were not properly recorded. It also failed to identify risk factors that lead to the development of cardiac diseases.

It is really important to develop a culture of early recognition and close follow-up, which can improve maternal tolerance to increase the cardiovascular burden that is part of the normal physiology in pregnancy. This will not only promote neonatal survival but also fetal growth. There should be separate and individual attention, along with counseling to women who get pregnant with a pre-existing heart disease. By identifying the risk factors and reducing the burden of cardiac disease in pregnancy, we can reduce maternal and fetal mortality.

## Conclusions

In our study, the most common cardiac disease among our patients was peripartum cardiomyopathy followed by rheumatic heart disease in women. However, since it is a small sample size, single-institute study, caution should be taken to apply its result to a broader population. A further large-scale trial is needed to understand cardiac disease in pregnant women in Pakistan. These cardiac diseases are responsible for fetal and maternal adverse outcomes. Every effort at the government, institution, and community levels should be made to create awareness regarding the pre-pregnancy counseling of women with cardiac disease. Every effort should be made to create awareness regarding pre-pregnancy counseling, so that associated fetal and maternal morbidity can be reduced.
